# Lymphatic Glands in the Cheek

**Published:** 1904-03-05

**Authors:** 


### LYMPHATIC GLANDS IN THE CHEEK.

Attention is called by Dr. M. Trendel to the presence of small lymphatic glands in the thickness of the cheek, which he believes to be of some clinical importance. The glands are arranged into three groups: (1) A maxillary group consisting of one or two glands which lie between the facial artery and vein at the anterior border of the masseter muscle; (2) a buccinator group, situated on the external surface of the buccinator muscle just below the level of a line drawn from the labial commissure to the lobule of the ear; (3) a superior maxillary group which lies in the naso-labial fold on a level with the lower border of the orbit at the internal palpebral angle. These glands may become infected and suppurate as the result of carious teeth, tonsillitis, erysipelas, and so forth. When this occurs the abscess nearly always opens, if untreated, on the external surface of the cheek. In cases of tubercle and malignant disease these glands may become affected and may be felt as hard nodules in the cheek, the best way to recognise them being to explore with one finger in the mouth while the other hand palpates the outer surface of the cheek. In every case of epithelioma of the face this examination should be carried out. The glands in question seldom exceed the size of a pea. If it is thought desirable to remove them the necessary incision should be made horizontally to avoid injury to the parotid duct and branches of the facial nerve.

